# Reliability of the Interprofessional Collaborator Assessment Rubric (ICAR) in Multi Source Feedback (MSF) with post-graduate medical residents

**DOI:** 10.1186/s12909-014-0279-9

**Published:** 2014-12-31

**Authors:** Mark F Hayward, Vernon Curran, Bryan Curtis, Henry Schulz, Sean Murphy

**Affiliations:** Patient Research Center, Faculty of Medicine, Memorial University, St. John’s, A1B 3 V6 NL Canada

**Keywords:** Interprofessional relations, Assessment, Multi-Source Feedback (MSF), Medical education

## Abstract

**Background:**

Increased attention on collaboration and teamwork competency development in medical education has raised the need for valid and reliable approaches to the assessment of collaboration competencies in post-graduate medical education. The purpose of this study was to evaluate the reliability of a modified Interprofessional Collaborator Assessment Rubric (ICAR) in a multi-source feedback (MSF) process for assessing post-graduate medical residents’ collaborator competencies.

**Methods:**

Post-graduate medical residents (n = 16) received ICAR assessments from three different rater groups (physicians, nurses and allied health professionals) over a four-week rotation. Internal consistency, inter-rater reliability, inter-group differences and relationship between rater characteristics and ICAR scores were analyzed using Cronbach’s alpha, one-way and two-way repeated measures ANOVA, and logistic regression.

**Results:**

Missing data decreased from 13.1% using daily assessments to 8.8% utilizing an MSF process, p = .032. High internal consistency measures were demonstrated for overall ICAR scores (α = .981) and individual assessment domains within the ICAR (α = .881 to .963). There were no significant differences between scores of physician, nurse, and allied health raters on collaborator competencies (F2,5 = 1.225, p = .297, η2 = .016). Rater gender was the only significant factor influencing scores with female raters scoring residents significantly lower than male raters (6.12 v. 6.82; F1,5 = 7.184, p = .008, η 2 = .045).

**Conclusion:**

The study findings suggest that the use of the modified ICAR in a MSF assessment process could be a feasible and reliable assessment approach to providing formative feedback to post-graduate medical residents on collaborator competencies.

## Background

In Canada, medical education at the undergraduate, post-graduate and continuing medical education (CME) levels is supported by the CanMEDS framework of the Royal College of Physicians and Surgeons of Canada [1]. The CanMEDS framework describes seven core roles that physicians should demonstrate competence in: Medical expert, Communicator, Collaborator, Manager, Health Advocate, Scholar, and Professional [1]. “Competency” has been defined as a dynamic concept that encompasses an understanding of the knowledge, clinical skills, interpersonal and problem solving skills required for excellence in professional performance [2]. The Collaborator role, in particular, defines physician collaboration as “*effectively working within a health care team to achieve optimal patient care”* [3]. The two key competencies for this role require that physicians are able to: 1) *Participate effectively and appropriately in an interprofessional health care team*; and 2) *Effectively work with other health professionals to prevent, negotiate and resolve interprofessional conflict*.

Competency-based education places a greater emphasis on the attainment of required competence and the practice of skills in the real environment. An essential principle of competency-based education is the ability to assess objectively for the achievement of competence. Massagli and Carline [4] suggest that physician competence is multi-dimensional and that no single tool is capable of assessing all aspects of competence. It has been recommended that assessment of the CanMEDS roles should be based upon a multi-faceted approach that occurs at varying times to assess different aspects of skill, attitude, behavior, and performance [3]. Many instruments have been reported for the self-assessment of attitudinal shifts, however there needs to be greater emphasis placed on the development of tools which rely on objective, external observer measurements of all types of competencies (knowledge, skills and attitudes) for interprofessional collaboration [3].

Multi-Source Feedback (MSF) has become a popular assessment process in medical education [5-7]. MSF, also known as 360-degree assessment, has been described as the use of specific processes and instruments for undertaking workplace-based assessment. Evidence on performance in the workplace can be collected from multiple sources, including senior colleagues, peers, nurses, other healthcare workers, and patients [8]. This method can be used for both formative and summative assessment purposes and has been widely used in postgraduate medical education [9,10], and CME [11]. MSF originated during the second World War and was adopted in industrial settings for employee performance evaluation and began to be adopted within healthcare in the late 1990’s [5,12]. As of 2009 over 4000 residency programs in North America and the UK report using MSF to assess residents and fellows [5]. MSF feasibility, reliability, and validity have been studied in various medical speciality programs including, but not limited to: Emergency Medicine [13], Internal Medicine [14], Obstetrics/Gynecology [15], Pathology [16], and Psychiatry [17].

Massagli and Carline [4] and Campbell et al. [18] note that MSF is best utilized when incorporated as part of a formative process of assessment whereby residents can review the results, or are provided feedback, to develop a plan of action to reach competency with their mentor or residency director. Systematic reviews of the literature on MSF concluded that incorporation of multiple perspectives in various environments is essential to evaluate performance [19-21]. Participating residents felt that the evaluations increased their awareness of how they interacted with patients [22]. When ratees take part in the evaluation process, it allows self-reflection, increased engagement in the evaluation process, and comparison as to how their self-assessment aligns with those they interact with. Similarly, it allows assessment from the perspectives of individuals who may rarely offer input, such as nurses, allied health professionals or even patients. Joshi et al. [15] demonstrated that in a stable institution, with a relatively small number of residents, MSF is a practical, effective evaluation of interpersonal and communication skills. More quantitatively, a systematic review by Donnon et al. [20] concluded that adequate statistical reliability and generalizability is achieved with the 41 participants: 8 medical peers, 8 non-physician co-workers (nurses, psychologists, pharmacists, and other allied health professionals) and 25 patients.

Hammock et al. [23] suggests MSF is an important mechanism for influencing the delivery of interprofessional education (IPE) by increasing awareness of the roles of other health professionals that contribute to quality patient care. Unfortunately, nursing staff are infrequently involved in resident evaluation as often it is only the attending physicians participating in completing surveys or questionnaires for a specific rotation [24]. Nursing staff may observe different aspects - such as team relationships, interactions with patients and family, and humanistic attitudes - of a resident’s performance that may not be viewed by attending physicians and thus may offer a unique perspective during resident assessment [24,25]. The ability of residents to create and maintain positive collaborative relationships with nursing staff is essential for patient safety and in establishing a mutually supportive clinical environment [6]. Studies have reported that physicians, faculty, nurses, and allied health professionals and patients can reliably rate physicians’ humanistic behavior [4,15,21]. Al Ansari et al. [21] conducted a meta-analysis that demonstrated acceptable construct validity using the MSF process for the assessment of physicians and surgeons across the multiple years of a residency, or in practice.

The Interprofessional Collaborator Assessment Rubric (ICAR) was originally developed for use in the assessment of interprofessional collaborator competencies [26]. The development of ICAR was guided by an interprofessional advisory committee comprising health professional educators from the fields of medicine, nursing and the rehabilitative sciences. The Rubric dimensions are based on interprofessional collaborator competency statements that were developed and validated through a typological analysis of national and international competency frameworks, a Delphi survey of experts, and interprofessional focus groups with students and faculty.

The purpose of the study was to evaluate the feasibility and reliability of the use of the ICAR in a MSF process for assessing post-graduate medical residents’ Collaborator competencies.

## Methods

### Instrument

The original version of the ICAR contains 31 evaluative items organized into 6 domains. Domains and associated items reflect competency statements of the Royal College of Physicians and Surgeons of Canada CanMEDS Collaborator role. Each item on the original ICAR is evaluated on a scale of 1 to 4 (1 = Minimal, 2 = Developing, 3 = Competent, 4 = Mastery) based on the frequency of demonstrated ability of the trainee as outlined by behavioral indicators. The content validity of the original ICAR version was reviewed with a small group of clinician-educators (MDs) with the purpose of affirming the relevance of items within a post-graduate medical education context. Items were removed or retained based on the level of agreement on each item between the reviewing physicians. From this review, a 17-item modified ICAR was pilot tested. The pilot study encompassed daily assessments (over a two week study period) of post-graduate trainees’ Collaborator competencies. The purpose of the pilot study was to evaluate the feasibility and inter-rater reliability of the ICAR. The pilot study results led to a modified revision of the ICAR in which the scoring scale was expanded to 9-points where “1 = well below expectations, 5 = meets expectations, and 9 = well above expectations” (http://www.med.mun.ca/CCHPE/Faculty-Resources/Interprofessional-Collaborator-Assessment-Rubric.aspx or: http://bit.ly/Rubric). The following methodological discussion pertains to the subsequent field test using the modified ICAR in a MSF assessment approach.

### Participants

Residents and medical staff from four post-graduate medical education programs (Internal Medicine, Obstetrics/Gynecology, Neurology, and Orthopedic Surgery) were recruited to participate in the field test study. Residents - from these disciplines - completed 4-week rotations on one of five medical/surgical units. Residents were excluded from the study if, during the assessment period, they were on a rotation, or elective, outside of the research hospital. Due to the inclusion/exclusion criteria of the study sixteen (n = 16) residents were deemed to be eligible to be assessed by attending physicians, nurses and allied professionals. Residents were blind to which rotation they would be evaluated on and which specific healthcare professionals were assessing them. To be eligible for inclusion in the final statistical analysis, a resident must have received a minimum of six (n = 6) assessments from at least two members in each rater group. In total, six (n = 6) residents met this requirement and were incorporated in the statistical analysis. The six residents were considered representative of the resident population of which we sampled from as the residents comprised at least four different medical disciplines covering each of the post-graduate years (PGY 1 – 5). Table 1 demonstrates the distribution of raters for each resident.

**Table 1:** **Chi-square analysis of rater distribution across residents**

	**Residents**							
	**A**	**B**	**C**	**D**	**E**	**F**		
**Rater group**							**Total**	***p***
Physicians	3	5	5	5	2	2	**22**	.202
Nurses	16	10	11	11	30	29	**107**	
Allied health	4	4	4	4	5	5	**26**	
**Total**	**23**	**19**	**20**	**20**	**37**	**36**	**155**	

Physicians, nurses and allied health professionals were recruited from the participating medical/surgical units to assess their respective residents on Collaborator competencies using the modified ICAR. Physicians were recruited based on the specific resident’s recommendations depending on which physicians they interacted with on their rotation. All nursing and allied health professionals on participating units were invited to complete an ICAR. Individuals from both groups were excluded if they missed at least one of the four weeks of the resident’s rotation. Division managers provided names and shift schedules for participating nurses and allied health professionals.

### Procedure

A cover page to the modified ICAR collected information on the descriptive characteristics of each rater, including: profession, gender, years of experience in profession, years of experience in current medical/surgical unit, frequency of interaction, and type of interaction. A direct interaction was defined as a ‘face-to-face or phone conversation’, while an indirect interaction was defined as ‘contact through chart notes, orders, or requests; discharge planning; hearing from other colleagues; or hearing from patient or family’. Descriptive characteristic variables for ‘years of experience in profession’, ‘years of experience in current medical/surgical unit’, and ‘frequency of interaction’ were transformed into new binary variables to allow adequate sample sizes for statistical analysis. Descriptive characteristics for each rater group and the distribution of raters per group across residents were compared using Pearson’s Chi-Square test.

Missing data ranged from 0% to 26.5% across the 17 items. All missing data in quantitative variables was replaced using a single imputation stochastic regression method [27]. This method imputed an individual missing value from the data set using the rater mean, item mean, grand mean, and a random error term.

Comparison of overall ICAR score was analyzed using one-way ANOVA for rater groups and the remaining binary descriptive characteristics. To determine the effect of independent variables on overall ICAR score, two-way repeated-measures ANOVA was utilized to test for within-subject and between-subject main effects and interactions across the 17 items of the ICAR between residents. A summary package of both quantitative and qualitative data was provided to the six residents involved in the analysis.

Ethics approval was received from the Interdisciplinary Committee on Ethics in Health Research (ICEHR), Memorial University of Newfoundland.

## Results and discussion

One hundred and five (n = 105) raters initially consented to participate. Of these, 80 raters completed an ICAR assessment form for a 76.2% response rate. One hundred and fifty-five (n = 155) assessments were completed indicating that each rater completed, on average, 1.94 (or ~2) ICAR assessments. The subsequent analysis was based on the completed 155 ICAR assessments of the six residents receiving at least two assessments per rater group.

Of the three participating professional groups, nurses and allied health professions had near equal response rates of 75.0% (n = 57) and 75.2% (n = 13) respectively. Physicians were found to have the highest response rate of 90.9% (n = 10). There was no significant difference in response rates between rater groups (χ^2^ = 0.19, *df* = 2, *p* = .909).

Table 1 summarizes the distribution of rater groups across residents. There was no significant difference in the proportion of raters per resident (χ^*2*^ = 13.412, *df* = 10, *p* = .202) with per resident raters ranging from 19 – 37. The ranges within rater groups across residents were: 2 – 5 physicians; 10 – 30 nurses; and 4 – 5 allied health professionals.

Table 2 summarizes the background characteristics of the rater groups. Nurses completed the majority of assessments (n = 107, 69.0%), followed by allied health professionals (n = 26, 16.8%), and physicians (n = 22, 14.2%). Females completed 81.3% (n = 126) of the total assessments. There were significant (*p* < .001) differences in the gender of participants from each rater group; male physicians (81.8%), female nurses (92.5%), and female allied health professionals (88.4%). There were more assessments completed by raters with at least 10 years of professional experience (60.0%) and in their current unit (55.5%). As well, the majority (65.8%) of assessments were completed by raters who reported at least one resident interaction per day.

**Table 2:** **Characteristics of rater groups**

	**Total**	**Physician**	**Nurse**	**Allied health**	**χ** ^***2***^	***p***
**Ratings (n, %)**	155	22 (14.2)	107 (69.0)	26 (16.8)		
**Gender**						
Female (n, %)	126 (81.3)	4 (18.2)	99 (92.5)	23 (88.5)	67.3	<.001*
Male (n, %)	29 (18.7)	18 (81.8)	8 (7.5)	3 (11.5)		
**Years in profession**						
<10 (n, %)	62 (40.0)	7 (31.8)	45 (42.1)	10 (38.5)	0.83	.660
10+ (n, %)	93 (60.0)	15 (68.2)	62 (57.9)	16 (61.5)		
**Years in current unit**						
<10 (n, %)	69 (44.5)	6 (27.3)	58 (54.2)	22 (84.6)	16.1	<.001*
10+ (n, %)	86 (55.5)	16 (72.7)	49 (45.8)	4 (15.4)		
**Interaction frequency**						
≥1 per shift (n, %)	102 (65.8)	15 (68.2)	80 (75.5)	7 (26.9)	22.1	<.001*
<1 per shift (n, %)	52 (33.5)	7 (31.8)	26 (24.5)	19 (73.1)		

*Significant at α <0.05.

A paired samples t-test analysis of missing data revealed a significant reduction between the MSF field test study using the ICAR and the initial pilot study, 8.8% vs. 13.1% respectively, p = .032 (Table 3). The final two items of the ICAR, #16 and #17 – both under the Conflict Management/Resolution domain – were found to have the highest percent of missing data in both the pilot and field test studies, averaging 22.3% and 40.6% respectively. The difference between means and standard deviation (SD) in the new and original dataset was −0.05 (6.30 vs. 6.25) and −0.04 (1.49 vs. 1.45) respectively. This result suggests that the replacement of missing data was successful in maintaining the validity of the data set and could be used for further analysis.

**Table 3:** **Comparison of missing data between pilot study and Multi-Source Feedback (MSF)**

**Item #**	**Item category (# in category)**	**Pilot (%)**	**MSF (%)**	**Difference**
**17**	Conflict Management/Resolution (3)	54.8	26.5	- 28.3
**16**	Conflict Management/Resolution (2)	25.8	18.7	- 7.1
**8**	Roles and Responsibility (1)	19.4	16.8	- 2.6
**10**	Roles and Responsibility (3)	19.4	15.5	- 3.9
**15**	Conflict Management/Resolution (1)	19.4	8.4	- 11.0
**12**	Patient/Client – Family Centred (2)	16.1	18.7	**+2.6**
**14**	Team Functioning (2)	16.1	3.9	- 12.2
**11**	Patient/Client – Family Centred (1)	12.9	17.4	**+4.5**
**9**	Roles and Responsibility (2)	9.7	7.1	- 2.6
**13**	Team Functioning (1)	9.7	5.8	- 3.9
**6**	Collaboration (2)	6.5	3.2	- 3.3
**2**	Communication (2)	3.2	1.3	- 1.9
**3**	Communication (3)	3.2	2.3	- 0.9
**5**	Collaboration (1)	3.2	3.2	0
**7**	Collaboration (3)	3.2	1.3	- 1.9
**1**	Communication (1)	0	0.6	**+0.6**
**4**	Communication (4)	0	0	0
	**Total Missing**	**13.1**	**8.8**	- 4.3*

*Significant at α = 0.05 (Paired samples t-test).

Table 4 summarizes internal consistency analyses of the modified ICAR and the associated domains of the instrument. An overall Cronbach’s alpha coefficient of α = .981 revealed high internal consistency reliability. Each domain also demonstrated high internal consistency, ranging between .881 - .963. Due to the high internal consistency of the domains, the overall ICAR scores used in further analysis were the sum of all 17 items from the six domains.

**Table 4:** **Internal consistency for modified ICAR competency domains**

**Competency domain**	**MSF** ^**‡**^
Communication (4 items)	.963*
Collaboration (3 items)	.950*
Roles and responsibility (3 items)	.899*
Collaborative patient/client – family centred (2 items)	.881*
Team functioning (2 items)	.932*
Conflict management/Resolution (2 items)	.907*
**ICAR (17 items)**	.981*

*> .70 indicates acceptable reliability.

^‡^MSF – Multi-Source Feedback.

**Figure 1:**
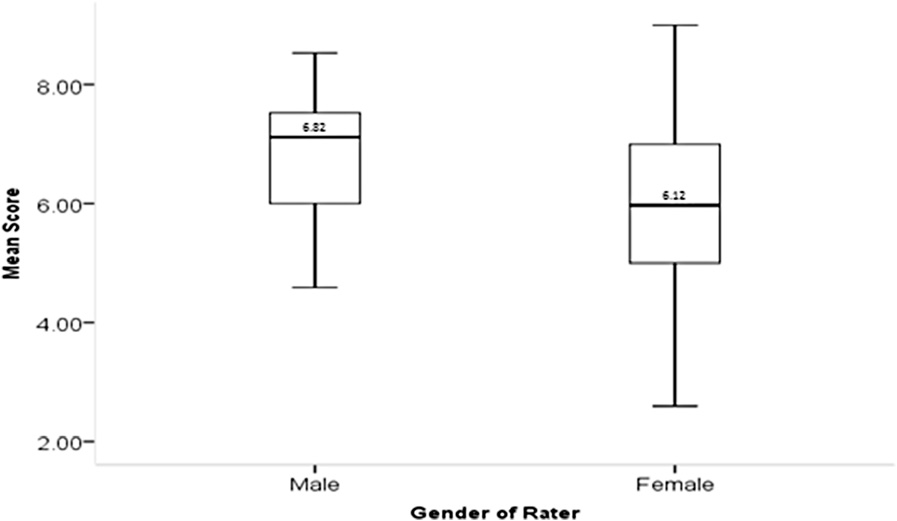
**Box plot of overall ICAR score difference between rater gender.**

Results of the ANOVA for determining which independent, or descriptive, variables of the rater’s background characteristics affected resident overall ICAR score are summarized in Table 5. The profession of the rater yielded no significant effect with a very small effect size (*F*_*2,5*_ = 1.225, *p* = .297, η^***2***^ = .016). The only significant, main-effect on overall ICAR score was found to be the gender of the rater (*F*_*1,5*_ = 7.184, *p* = .008, η^***2***^ = .045) providing a moderate effect size constituting 4.5% of the variance. Female raters scored residents significantly lower than male raters (6.12 v. 6.82). Figure 1 depicts the significant difference between male and female rater overall ICAR scores.

**Table 5:** **One-way ANOVA of overall ICAR scores by rater characteristics**

		**ICAR scores**				
	**N**	**Overall** ^**α,β**^	**s**	**F**	***p***	**η** ^***2***^
**Profession**				1.225	.297	.016
Physician	22	6.64	1.13			
Nurse	107	6.21	1.34			
Allied health	26	6.09	1.30			
**Gender of rater**				7.184	.008*	.045
Female	126	6.12	1.03			
Male	29	6.82	1.33			
**Years in profession**				0.949	.331	.006
<10	62	6.12	1.27			
10+	93	6.33	1.32			
**Years in current unit**				0.011	.917	.000
<10	86	6.24	1.29			
10+	69	6.26	1.33			
**Interaction frequency**				0.310	.579	.002
≥1 per shift	102	6.30	1.35			
<1 per shift	52	6.18	1.22			
**Gender of Resident**				0.013	.908	.000
Female	2	6.23	1.34			
Male	4	6.26	1.29			

^**α**^Overall ICAR score determined by summing total score divided by total number of raters.

^**β**^ICAR scored on a 9-point scale.

^*^Significant at α = 0.05.

**Figure 2:**
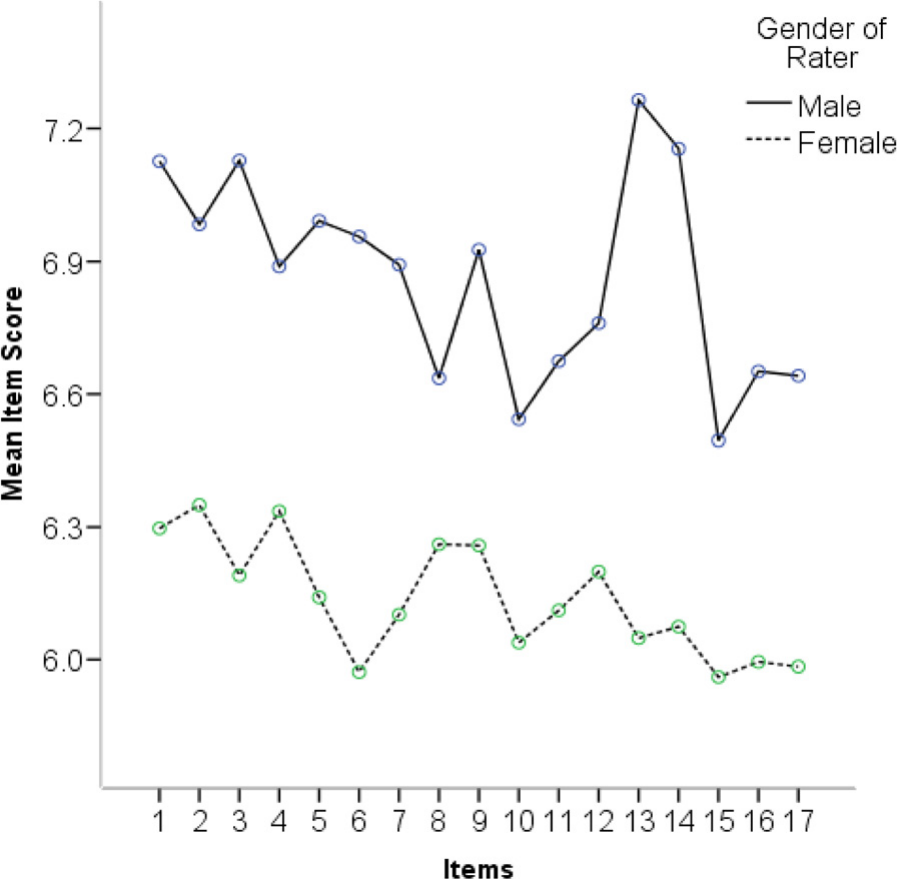
**Mean rater scores across ICAR items for male and female raters.**

A further gender effect was found through two-way repeated measures ANOVA analysis that revealed a significant interaction effect (F = 1.911, p = .021, η^**2**^ = .013) of rater gender across the 17 item scores. Figure 2 depicts the overall ICAR scores across the 17 items for male and female raters. It is important to note that there was no interaction effect between resident gender and rater gender, *p = .359*.

A final analysis underscoring the effect gender played on overall ICAR score utilized logistic regression. The analysis revealed that rater gender was the only significant predictor of overall ICAR score. Male raters were 3.08 times more likely than female raters to provide an overall ICAR score of above 6.0 (p = .013) and 3.28 times more likely to score above 7.0 (p = .005).

**Figure 3:**
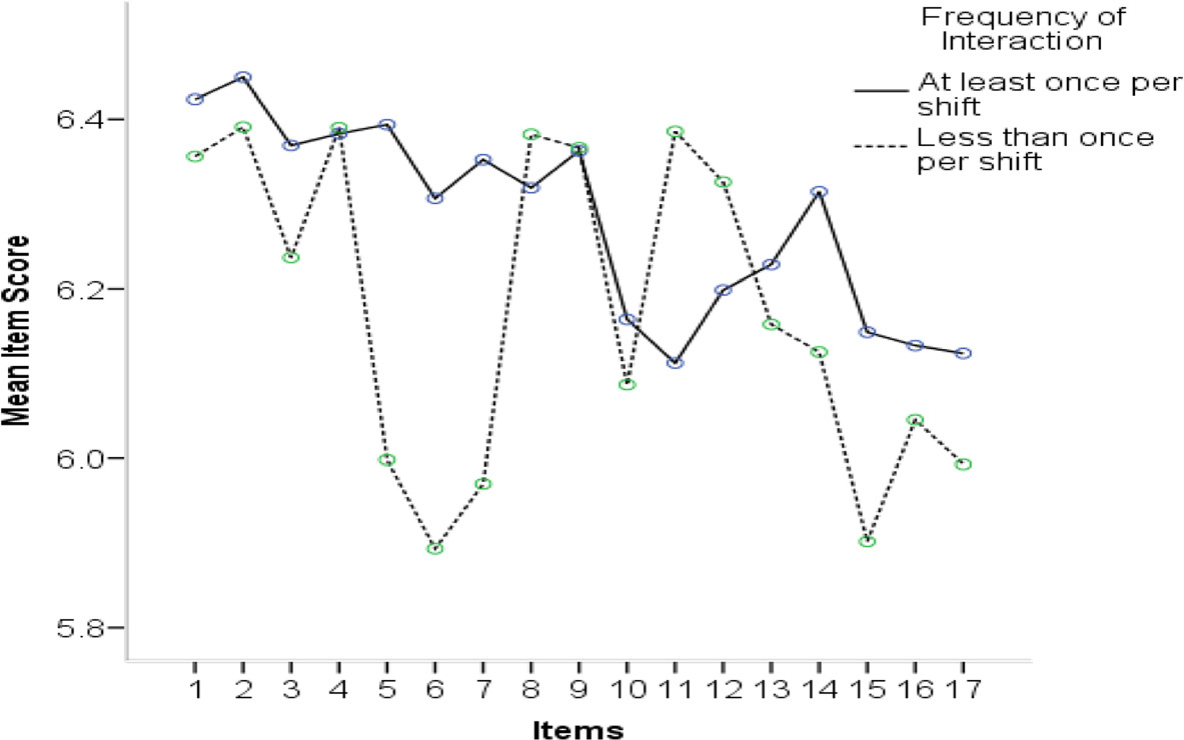
**Overall ICAR score by interaction frequency.**

A significant interaction effect resulted from a two-way repeated measures ANOVA analysis involving the frequency of interaction between raters and residents across items (F = 2.103, p = .025, η ^**2**^ = .014). The post-hoc analysis revealed effect was due to items #5, 6 and 7 of the ICAR (all comprising the ‘Collaborator’ domain). Figure 3 depicts the overall ICAR score for items #5, #6, and #7 being scored lower by raters who interacted with residents less than once per shift.

A significant difference was found between overall ICAR scores for each of the 17 items. Analysis revealed there was a significant main effect on the means of the individual 17 items (*F* = 2.79, *p* = .002, η ^***2***^ = .02), indicating a small effect size accounting for 2% of the total variance. However, rater groups did not differ in their scores across items as indicated by a non-significant interaction effect (*F* = 0.807, *p* = .713, η ^***2***^ = .012).

Finally, qualitative data was recorded to supplement the quantitative scores. A summary package of both quantitative and qualitative data was provided to the six residents involved in the analysis. The qualitative data illustrates the variety of feedback that can be received. For example one resident received positive (*Good communication skills. Able to amalgamate clinical knowledge to these scenarios*), neutral (*Very rare for this specific rater to have interactions with this resident. But when on unit, zero problems or issues with collaboration*), and negative (*Would rather do anything but listen to suggestions of those he feels are below him. A smart student but should be more respectful of the interdisciplinary team*) feedback.

## Discussion

The overall response rate (76.2%) in the field test of the modified ICAR in a MSF assessment process was generally high for all rater groups; ranging from 75.0% to 90.9%. This result reflects the upper end of response rates reported in the literature regarding MSF feedback which ranges from 36% [28] to 95% [17]. This response rate suggests that the use of the ICAR in a MSF process with post-graduate residents may be a viable option to assess Collaboration competencies. The modified ICAR also demonstrated high internal consistency reliability for the overall ICAR score and each of the domains (α = .881 - .963). A reduction in missing data between the pilot and field test of the modified ICAR suggests that prolonged observation periods may be needed for adequate assessment of Collaborator competencies. Items in the ‘Conflict Resolution and Management’ domain also demonstrated a high proportion of missing data despite the extended observation period in the field test. Conflict resolution and management skills may be more challenging competencies to assess through direct observation, particularly if the work environment is well functioning and highly productive.

Analysis of overall ICAR scores revealed no significant differences between physicians, nurses, and allied health professionals. This finding tends to support the inter-rater reliability of the modified ICAR form and its use in a MSF assessment process. This result may also counter claims that non-physician medical staff are unable to provide reliable observations of non-medical expert roles such as Collaborator competencies. In earlier work, Rezler et al. [29] reported that some residents had questioned whether nurses or allied health professionals had the ability to evaluate them adequately and Canavan et al. [30] found that feedback from nursing and allied health professionals was overly positive and useful to enhancing performance improvement in particular areas. The MSF literature suggests that MSF is best used as a form of formative assessment and feedback [10].

The analysis did reveal significant findings with respect to the gender of the rater, but not the gender of the resident. Male raters tended to rate residents more highly than female raters. It is difficult to infer from these results whether female raters had higher expectations (e.g., score lower) than males with respect to the Collaborator competencies of the residents. Earlier work has indicated significant differences in gender attitudes towards interprofessional healthcare teams [31,32]. Ostroff et al. [33] has also examined the predictive ability of background characteristics on the score an individual would receive and found that male raters tended to be over-estimators of an individual’s performance. Analysis of the gender of residents did not yield a significant difference in overall mean ICAR score which is contrary to other findings. Previous research has suggested that female medical learners score higher than their fellow male students [34-38].

The qualitative data demonstrated rich value for the medical learners as they were able to not only see their collaborative abilities as a number but also how it affected the team they worked with. The anonymous feedback provided a variety of constructive feedback from positive, neutral, and negative responses from which the learner can reflect on. The participating residents were quite appreciative to receive the qualitative feedback.

The main limitations of the study were that it was conducted in a single institution and on only four medical units. The sample sizes of physicians and allied health professionals were also low. It was not possible to recruit the adequate participants to meet the criteria denoted by Donnon et al. [20] of 41 participants: 8 physicians, 8 coworkers, and 25 patients given the time limitation and the non-participation of patients. There was also an uneven distribution of resident gender and residents indicated which physicians were appropriate to assess them.

## Conclusion

The study findings suggest that the use of the modified ICAR form in a MSF assessment process could be a feasible assessment approach to providing formative feedback to post-graduate medical residents on Collaborator competencies. There were no significant differences in the overall mean ICAR score between three interprofessional rater groups across three different medical units. The experience level of the rater and the frequency of interaction with the resident also had no significant effect on the overall ICAR score. Qualitative data demonstrated the array of feedback that can be provided to learners, which was appreciated by the participants.

## Abbreviations

CME: Continuing medical education
ICAR: Interprofessional collaboration assessment rubric
IPE: Interprofessional education
MSF: Multi-source feedback
PGY: Post-graduate year
